# Reduction in *Clostridium difficile* Infection Rates after Mandatory Hospital Public Reporting: Findings from a Longitudinal Cohort Study in Canada

**DOI:** 10.1371/journal.pmed.1001268

**Published:** 2012-07-17

**Authors:** Nick Daneman, Therese A. Stukel, Xiaomu Ma, Marian Vermeulen, Astrid Guttmann

**Affiliations:** 1 Institute for Clinical Evaluative Sciences, Toronto, Ontario, Canada; 2 Division of Infectious Diseases, Department of Medicine, Sunnybrook Health Sciences Centre, University of Toronto, Toronto, Ontario, Canada; 3 Institute of Health Policy, Management and Evaluation, University of Toronto, Toronto, Ontario, Canada; 4 Division of Paediatric Medicine, Hospital for Sick Children, Toronto, Ontario, Canada; 5 Department of Paediatrics, University of Toronto, Ontario, Canada; University of Geneva Hospitals and Medical School, Switzerland

## Abstract

**Background:**

The role of public reporting in improving hospital quality of care is controversial. Reporting of hospital-acquired infection rates has been introduced in multiple health care systems, but its relationship to infection rates has been understudied. Our objective was to determine whether mandatory public reporting by hospitals is associated with a reduction in hospital rates of *Clostridium difficile* infection.

**Methods and Findings:**

We conducted a longitudinal, population-based cohort study in Ontario (Canada's largest province) between April 1, 2002, and March 31, 2010. We included all patients (>1 y old) admitted to 180 acute care hospitals. Using Poisson regression, we developed a model to predict hospital- and age-specific monthly rates of *C. difficile* disease per 10,000 patient-days prior to introduction of public reporting on September 1, 2008. We then compared observed monthly rates of *C. difficile* infection in the post-intervention period with rates predicted by the pre-intervention predictive model. In the pre-intervention period there were 33,634 cases of *C. difficile* infection during 39,221,113 hospital days, with rates increasing from 7.01 per 10,000 patient-days in 2002 to 10.79 in 2007. In the first calendar year after the introduction of public reporting, there was a decline in observed rates of *C. difficile* colitis in Ontario to 8.92 cases per 10,000 patient-days, which was significantly lower than the predicted rate of 12.16 (95% CI 11.35–13.04) cases per 10,000 patient-days (*p*<0.001). Over this period, public reporting was associated with a 26.7% (95% CI 21.4%–31.6%) reduction in *C. difficile* cases, or a projected 1,970 cases averted per year (95% CI 1,476–2,500). The effect was specific to *C. difficile*, with rates of community-acquired gastrointestinal infections and urinary tract infections unchanged. A limitation of our study is that this observational study design cannot rule out the influence of unmeasured temporal confounders.

**Conclusions:**

Public reporting of hospital *C. difficile* rates was associated with a substantial reduction in the population burden of this infection. Future research will be required to discern the direct mechanism by which *C. difficile* infection rates may have been reduced in response to public reporting.

*Please see later in the article for the Editors' Summary*

## Abstract 2

A population-based study conducted by Nick Daneman and colleagues in Ontario, Canada reports on the association between population reporting of hospital infection rates and a reduction in population burden of *Clostridium difficile* colitis.

## Editors' Summary

**Background:**

A stay in hospital can be lifesaving but can expose people to health care–associated infections. One of these—*Clostridium difficile* infection—is a major cause of infectious disease illness and death in developed countries. *C. difficile* bacteria cause diarrhea and, more rarely, life-threatening inflammation of the gut (colitis). They are present in the gut of about 3% of adults but do not normally cause any problems because other “good” bacteria keep them in check. However, antibiotics destroy these good bacteria, and if a person who has taken antibiotics becomes infected with *C. difficile* before good bacteria repopulate the gut, *C. difficile* can multiply rapidly and produce toxins that cause illness. Because *C. difficile* is usually acquired from other infected patients and their contaminated environments, and because antibiotic use is highly prevalent in hospitals, most *C. difficile* infections are acquired in hospitals and nursing homes. Infections can be prevented by practicing good hygiene in health care environments (for example, washing hands regularly with soap and water), by isolating patients who are infected with *C. difficile*, and by prescribing antibiotics for other infections sparingly.

**Why Was This Study Done?:**

Hospitals often need encouragement to improve infection control and other aspects of care. One potential way to improve the quality of hospital care is mandatory public reporting of measures of care quality. This intervention may help hospitals identify areas of poor performance to target for improvement or may motivate them to improve care quality to avoid the shame of a bad performance report. Although many health care systems have introduced public reporting of hospital-acquired infections, the effects of this intervention have been poorly studied. In this longitudinal cohort study, the researchers use population-based health care data to evaluate the impact of the introduction of mandatory hospital public reporting of the rates of hospital-acquired *C. difficile* infection in Ontario, Canada. Since September 1, 2008, hospitals in Ontario have been required to send monthly data on hospital-acquired *C. difficile* infections to the Ontario Ministry of Health and Long-Term Care for posting on a public website.

**What Did the Researchers Do and Find?:**

The researchers used health care administrative data for all patients older than one year admitted to acute care hospitals in Ontario between April 1, 2002, and March 31, 2010, to develop a model to predict monthly rates of *C. difficile* disease per 10,000 patient-days based on rates in the period before the introduction of public reporting. They then compared the observed rates of *C. difficile* disease after the introduction of public reporting with the rates predicted by this model. In the pre-intervention period, there were nearly 34,000 cases of *C. difficile* disease during about 39 million hospital days. Rates of *C. difficile* disease increased from 7.01 cases per 10,000 patient-days in 2002 to 10.79 cases per 10,000 patient-days in 2007. After the introduction of public reporting, the *C. difficile* disease rate fell to 8.92 cases per 10,000 patient-days, which is significantly (that is, unlikely to have occurred by chance) lower than the 12.16 cases per 10,000 patient-days predicted by the pre-intervention model. Finally, the researchers estimate that public reporting was associated with a 26.6% reduction in *C. difficile* disease cases and that it averted about 1,900 cases per year.

**What Do These Findings Mean?:**

These findings suggest that mandatory public reporting of hospital rates of *C. difficile* disease may reduce the population burden of this serious infection. Because this is an observational study, these findings do not prove that the introduction of mandatory public reporting actually caused a reduction in infection rates. Some other uncharacterized factor might be responsible for the decrease in *C. difficile* disease in Ontario hospitals since late 2008. Moreover, the many assumptions included in the predictive model means that the estimated number of cases averted by the introduction of public reporting may be inaccurate. Although further research is needed to determine how public reporting might affect *C. difficile* disease rates, the researchers suggest that, in this study, mandatory public reporting may have increased the prominence of *C. difficile* on hospital quality improvement agendas and may have motivated hospitals to adhere more closely to best practices in *C. difficile* prevention.

**Additional Information:**

Please access these websites via the online version of this summary at http://dx.doi.org/10.1371/journal.pmed.1001268.

- The US Centers for Disease Control and Prevention provides detailed information about *C. difficile* infection, including an article called Making Health Care Safer: Stopping *C. difficile* Infections
- The UK National Health Service Choices website provides information about *C. difficile* infections
- The Health Protection Agency provides information about mandatory reporting of *C. difficile* infections in England and Wales and a fact sheet on *C. difficile*
- Information about public reporting of hospital *C. difficile* rates in Ontario is available (in English and French)
- MedlinePlus provides links to further resources about *C. difficile* infections (in English and Spanish)
- The UK Clostridium Difficle Support website has a forum containing personal stories about *C. difficile* infection

## Introduction

The past decade has witnessed a remarkable global surge in the incidence and severity of *Clostridium difficile* infection [1]–[4]. This pathogen has long been among the most burdensome hospital-acquired infections, but has now become a leading contributor to infectious disease morbidity and mortality in developed countries [5]. The majority of *C. difficile* infections are acquired in hospital, and many of these are potentially preventable through strategies aimed at minimizing transmission between patients or decreasing patient susceptibility, primarily by reducing inappropriate antibiotic use [6].

Mandating public reporting of quality care metrics has been one vehicle used by health care payers to incentivize improvements in hospital care at a system level. Several theories have been put forth for how public reporting might improve patient outcomes: public reporting could encourage patients and their agents to select higher performing institutions (selection pathway), it could allow hospitals to identify areas of poor performance to target for improvement (change pathway), or it could motivate institutions to avoid the shame of a bad performance report and seek the pride of a good report (reputation pathway) [7],[8]. Most evidence, including a recent randomized trial of public reporting of cardiac quality indicators, suggests that the potential benefit is largely in stimulating quality improvement efforts [9]. However, the benefits and hazards of public reporting systems may vary depending on the specific clinical context (identification of appropriate measurements and responsible providers, and the need for risk adjustment) [7],[10],[11], and public reporting is particularly understudied in the domain of hospital-acquired infections [12].

In Ontario, Canada, the Ontario Ministry of Health and Long-Term Care (MOHLTC) selected *C. difficile* rates as the first among a slate of hospital patient safety indicators to be subject to mandatory monthly public reporting starting September 1, 2008 [13]. Hospitals were obligated to collect their own data on hospital-acquired *C. difficile* infections, and report the data monthly to the MOHLTC for posting on a publicly accessible website. No additional hospital-level incentives or disincentives were initially implemented, but there was acknowledgment that hospital global budgets and hospital executive compensation could eventually be linked to hospital performance [14].

The objective of our investigation was to utilize population-based health care data in Ontario, Canada, as an independent means to rigorously evaluate the impact of a public reporting system on province-wide, hospital-specific rates of this burdensome pathogen.

## Methods

### Ethics Statement

The study was approved by the research ethics board of Sunnybrook Health Sciences Centre.

### General Study Design

We conducted a retrospective, longitudinal population-based cohort study of all patients (>1 y old) admitted to acute care hospitals in Ontario between April 1, 2002, and March 31, 2010. Poisson regression analysis was used to develop a high-fidelity predictive model of hospital rates of *C. difficile* infection prior to public reporting, to allow an assessment of the change in provincial hospital-specific rates of *C. difficile* infection following the introduction of mandatory public reporting (introduced September 2008).

### Data Sources

The cohort was derived from a linkage of well-validated, province-wide health care administrative databases housed at the Institute for Clinical Evaluative Sciences [15],[16]. The Registered Persons Database contains demographic data for all of Ontario's 12.2 million residents; the Ontario Health Insurance Plan database includes physician billing claims for all visits and procedures performed within Ontario's universal single-payer health care system; the Canadian Institute for Health Information Discharge Abstract Database details all hospitalization events; the Ontario Drug Benefit database contains comprehensive and accurate (>99% concordant with chart review) outpatient drug information for Ontario's 1.2 million elderly residents [16].

*C. difficile* rates from health care administrative data were validated against rates reported by individual institutions via the mandatory public reporting system. Given that these reporting data are only available (by definition) from the period after the program was initiated, and given that they are aggregated at the hospital level (without any risk adjustment), they cannot be directly used to evaluate the impact of the program. Hospitals reported all cases of *C. difficile* diagnosed at their institution, using a standardized case definition, and were required to classify the origin of cases as (i) nosocomial acquisition from their institution, (ii) nosocomial acquisition from another institution, or (iii) community acquisition or unknown/indeterminate source. Only those cases deemed by the institution to have been acquired in their own hospital are incorporated into the numerator of monthly publicly reported rates. However, for this study we had access to overall *C. difficile* rates, including cases assigned to any of the three categories.

### Patient Selection Criteria

We identified all patients admitted to an acute care hospital in Ontario between April 1, 2002, and March 31, 2010. We excluded infants as well as patients admitted to psychiatry, rehabilitation, and complex continuing care institutions, given low expected event rates, and the fact that admissions to these non-acute-care centers are recorded in separate, less well validated databases. The use of broad inclusion criteria was intended to generate population-based data, and avoid the selection bias that is inherent in clinical surveillance networks. In total, data from 180 acute care hospitals contributed to the analysis during the 8-y study period, including 165 which contributed data to the public reporting period (September 1, 2008, to March 31, 2010). Individual hospitals were included even if they were in existence for only part of the pre-intervention and/or post-intervention period(s), because even with hospital openings, closures, and mergers during the 9-y study period, the net population at risk was considered to be all Ontario patients admitted to acute care hospital beds.

### Primary Predictor

The primary predictor in this study was the date of initiation of mandatory public reporting of hospital *C. difficile* rates, which was implemented by the MOHLTC on September 1, 2008.

### Outcome Definition

The primary outcome was the hospital- and age-specific monthly rates of *C. difficile* disease per 10,000 hospital patient-days. *C. difficile* disease is captured in the hospital database, through a single *International Classification of Diseases* (ICD) code (ICD-10 code A047). Two prior validation studies in the United States have indicated that ICD codes are sensitive (71%–88%) and specific (>99%) for the diagnosis of *C. difficile* infection [17],[18]. It is less clear whether administrative databases can accurately distinguish the source of *C. difficile* acquisition, and so we included all cases rather than just those that were labelled as post-admission diagnoses [19].

### Validation of *C. difficile* Rates in the Administrative Datasets

For additional indirect validation of ICD-10 codes in the local context, data from September 1, 2008, to March 31, 2010 (the period of public reporting), were used to compare hospital-level *C. difficile* counts in the administrative data to publicly reported counts. Pearson correlation coefficients (weighted for hospital bed-days) were calculated for overall *C. difficile* cases and nosocomial *C. difficile* cases across the institutions subject to public reporting.

### Risk Strata

Hospitalized patients were grouped into monthly, age group, and hospital-specific strata. Hospitalized patients were separated into nine age strata: 1–18, 19–30, 31–40, 41–50, 51–60, 61–70, 71–80, 81–90, and >90 y old. In total there were 124,740 potential strata in the pre-intervention period (77 mo×nine age groups×180 institutions), and 30,780 potential strata in the post-intervention period (19 mo×nine age groups×180 institutions). The actual number of strata was slightly lower (133,418), given that not all age strata were represented in all hospital-months, and some hospitals were not in existence for the entire study duration.

### Statistical Analysis

#### Primary analysis

We first computed *C. difficile* rates by age group, month, and hospital strata prior to September 1, 2008, to visually inspect temporal trends, overall and by hospital. The numerator was the number of *C. difficile* cases in each stratum; the denominator was the at-risk hospitalized population in each stratum (10,000 patient-days). In the primary analysis, we examined whether the introduction of public reporting of *C. difficile* rates in September 2008 was associated with a significant decline in hospital-specific *C. difficile* rates.

To model the temporal patterns of *C. difficile* infections, we used generalized estimating equations for clustered count data to account for correlations among outcomes within hospitals over time, using an auto-regressive correlation structure with a period of 3 mo [20]. The unit of analysis was the hospital, month, and age group stratum. The dependent variable was the number of *C. difficile* infections in each stratum; the offset parameter was the number of patient-days in each stratum. All models included age group, pre-reporting calendar month (to account for seasonal trends), hospital facility type (acute teaching, large community, or small community), concurrent and 1- to 12-mo lagged provincial monthly rates of prescriptions of antimicrobials, and indicator variables for each post-reporting month, coded as the difference between the specific post-reporting month and the corresponding pre-reporting calendar month. The exponentiated post-month regression coefficients thus represent the relative difference between the observed post-month and the predicted post-month based on the pre-reporting trends. We planned to model longitudinal trend with both linear and non-linear functions. Model fit was assessed through graphical inspection of observed and predicted rates over the pre-intervention period, as well as through deviance and Pearson chi-square statistics.

We used 6.5 y of data to model pre-reporting trends, and projected these trends to the post-reporting period after September 1, 2008, to obtain predicted rates in the absence of reporting. The predicted cases and 95% confidence intervals for a calendar month after public reporting were computed as the observed number of cases for that month divided by the corresponding relative rates from the model, as in previous work [21]. The overall relative difference in infection rates in the calendar year 2009 was obtained as the exponentiated weighted average of the regression coefficients corresponding to that period, weighting by log person-days. Averted cases were computed as the difference between predicted and observed cases in post-reporting months. The decrease in hospital *C. difficile* rates was computed for the overall population, as well as for hospital facility subtypes. In a sensitivity analysis, to test the robustness of our results and to ensure that findings were not being driven by coding practices in a few large institutions, we excluded hospitals with discordant rates of *C. difficile* in the public reporting and administrative datasets.

#### Tracer analyses

To test the specificity of our findings, we examined tracer outcomes that should not be impacted by *C. difficile* public reporting. First, we inspected hospital admissions for other bacterial gastrointestinal pathogens. These pathogens (*Salmonella*, *Shigella*, *Campylobacter*, *Listeria*, and *Yersinia*, among others) are almost universally community-acquired, to the extent that microbiology laboratory guidelines recommend against testing for these pathogens when infections arise more than 72 h post-admission [22]. We also inspected urinary tract infections as a tracer outcome; although a fraction of urinary tract infections are hospital-acquired, they are not yet subject to public reporting, and the primary means of preventing nosocomial urinary tract infections (e.g., urinary catheter avoidance) differ from the prevention strategies for *C. difficile* [6],[23].

Analyses were performed using STATA procedure XTGEE. Patient confidentiality was maintained via encrypted health card numbers using Institute for Clinical Evaluative Sciences protocols.

## Results

### Cross-Validation of *C. difficile* Rates in Public Reporting and Administrative Datasets

**Figure 1:**
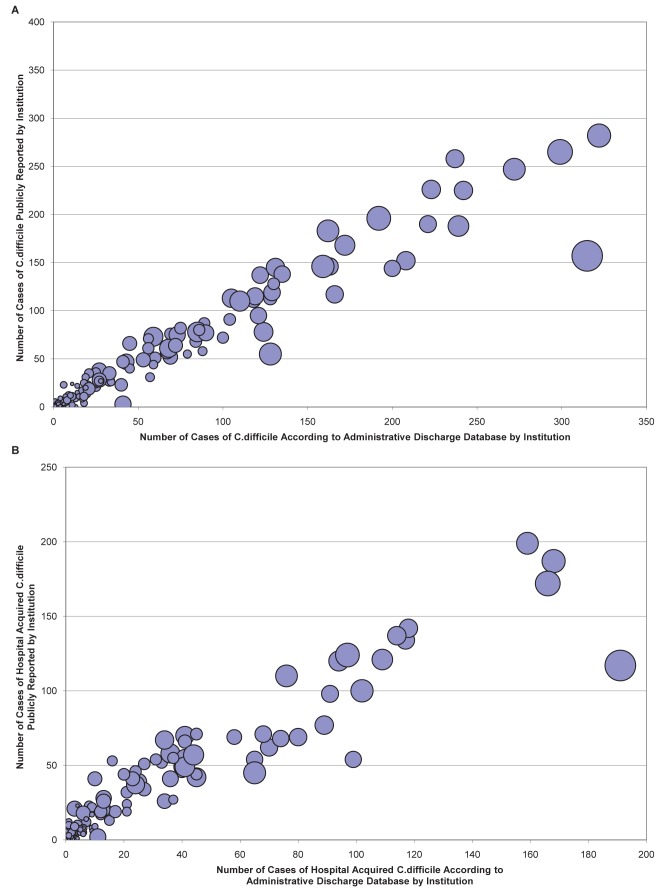
Correlation of aggregate hospital *C. difficile* cases in administrative datasets and public reporting statistics. *C. difficile* cases in the administrative data are plotted against cases from the public reporting database, for the total public reporting period between September 1, 2008, and March 31, 2010. Each bubble represents a distinct institution (*n* = 165), and bubble sizes reflect hospital sizes (in patient-days). There was an excellent correlation for overall *C. difficile* cases (A) (weighted Pearson's correlation coefficient 0.92), and hospital-acquired *C. difficile* cases (B) (weighted Pearson's correlation coefficient 0.91).

The total number of *C. difficile* cases publicly reported by each Ontario hospital between September 1, 2008, and March 31, 2010, was compared to the corresponding number of cases recorded in the provincial administrative datasets. There was an excellent concordance for overall *C. difficile* cases (Figure 1A; weighted Pearson's correlation coefficient 0.92), and nosocomial *C. difficile* cases (Figure 1B; weighted Pearson's correlation coefficient 0.91) across these institutions.

### *C. difficile* Rates in Ontario prior to the Introduction of Public Reporting

There were a total of 6,068,777 acute care hospital admissions, and 39,221,113 hospital days at risk for *C. difficile* infection in the pre-intervention period between April 1, 2002, and August 31, 2008. During this time there were 33,634 cases of *C. difficile* infection, corresponding to a rate of 5.54 per 1,000 hospitalizations or 8.58 per 10,000 patient-days. Of these cases, 14,956 (44.5%) were recorded as post-admission complications, corresponding to a rate of 2.46 nosocomial cases per 1,000 hospitalizations or 3.81 per 10,000 patient-days.

Provincial monthly rates per 10,000 patient-days did not differ by sex (data not shown), but did increase markedly in older age groups: 4.86 infections per 10,000 patient-days for 1–18 y olds, 2.88 for 19–30, 3.31 for 31–40, 5.84 for 41–50, 6.97 for 51–60, 8.55 for 61–70, 10.84 for 71–80, 12.05 for 81–90, and 12.46 for >90 y olds. *C. difficile* rates were highest in large community hospitals (8.97 per 10,000 patient-days), followed by academic teaching hospitals (8.01) and small community hospitals (7.88).

**Figure 2:**
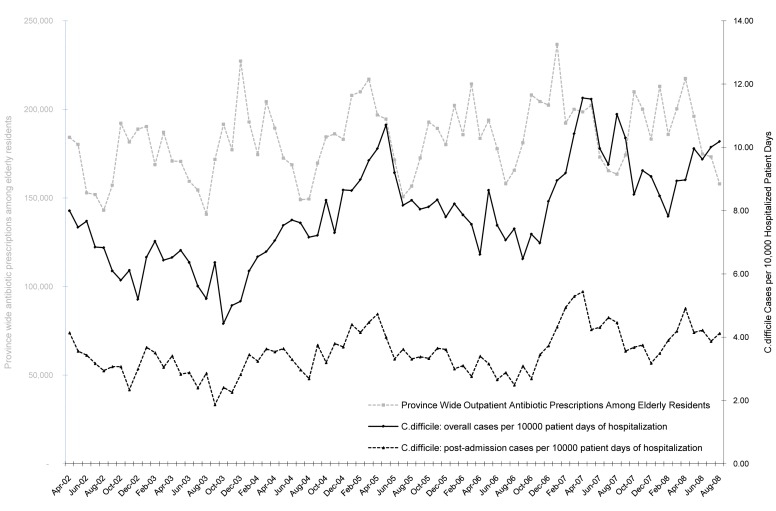
Longitudinal trends in *C. difficile* infection rates and antibiotic prescription rates in Ontario prior to the introduction of mandatory public reporting. Seasonal variations in overall *C. difficile* infection rates (black solid line) and post-admission *C. difficile* infection rates (black dashed line) per 10,000 patient-days appear to follow seasonal changes in the overall monthly population burden of antibiotic prescriptions measured by the number of prescriptions in the Ontario Drug Benefit database (grey dashed line).

There was marked seasonal variation in *C. difficile* rates, with yearly peaks in winter months (Figure 2). In addition, crude *C. difficile* rates increased over this pre-intervention period, from 7.01 per 10,000 patient-days in 2002, to 6.39 in 2003, 8.14 in 2004, 9.5 in 2005, 8.23 in 2006, and 10.79 in 2007. The overall burden of antibiotic use in Ontario (as measured by outpatient antibiotic prescriptions to elderly individuals) also exhibited seasonal peaks preceding *C. difficile* peaks, and increased over the pre-intervention period (Figure 2). The yearly antibiotic consumption increased from 2,110,184 prescriptions in 2003, to 2,323,006 in 2008, and 2,426,138 in 2009. The final predictive model incorporated age, hospital type, calendar month, and burden of antibiotic use in current and 12 lagged months. In this model there was no statistically significant longitudinal time trend prior to public reporting (the increase in pre-intervention rates was predicted by the combination of other covariates).

### Predictors of Monthly *C. difficile* Rates

As compared to the reference age group of 61–70 y olds, all younger age groups had a reduced incidence rate ratio of *C. difficile* infection, while all older age groups had an increased incidence (Table 1). Infection rates were lower in small community hospitals, as compared to academic teaching hospitals. Higher *C. difficile* monthly rates were associated with higher total outpatient elderly antibiotic prescriptions in Ontario in the preceding months, with time lags up to 9 mo (Table 1).

**Table 1:** Predictors of monthly hospital-specific *C. difficile* infection rates in a multivariate Poisson model of the period prior to public reporting.

Predictor	Rate Ratio	95% Confidence Interval
**Age group** a		
1–18 y old	0.57	0.34–0.95
19–30 y old	0.34	0.29–0.40
31–40 y old	0.39	0.33–0.46
41–50 y old	0.69	0.61–0.78
51–60 y old	0.81	0.73–0.91
71–80 y old	1.27	1.14–1.41
81–90 y old	1.39	1.25–1.55
>90 y old	1.43	1.25–1.63
**Hospital type** b		
Large community	1.03	0.94–1.12
Small community	0.83	0.71–0.96
**Total elderly outpatient antibiotic prescriptions**		
Current month	1.03	0.90–1.17
1 mo prior	1.43	1.26–1.61
2 mo prior	1.35	1.19–1.54
3 mo prior	1.22	1.08–1.39
4 mo prior	0.98	0.86–1.11
5 mo prior	1.15	1.00–1.31
6 mo prior	1.26	1.09–1.46
7 mo prior	1.20	1.03–1.40
8 mo prior	1.22	1.07–1.39
9 mo prior	1.35	1.16–1.57
10 mo prior	1.10	0.94–1.27
11 mo prior	1.11	0.98–1.27
12 mo prior	0.90	0.78–1.04
**Calendar month** c		
January	1.07	0.91–1.27
February	1.11	0.96–1.29
March	1.21	1.03–1.41
April	1.23	1.07–1.41
May	1.15	1.02–1.30
June	0.98	0.89–1.07
August	1.05	0.96–1.14
September	1.04	0.93–1.15
October	1.02	0.89–1.17
November	0.96	0.83–1.12
December	0.99	0.85–1.14

a Compared to 61–70 y old reference age group.

b Compared to academic teaching hospital reference group.

c Compared to July as a reference standard.

### *C. difficile* Rates in Ontario following the Introduction of Public Reporting

**Figure 3:**
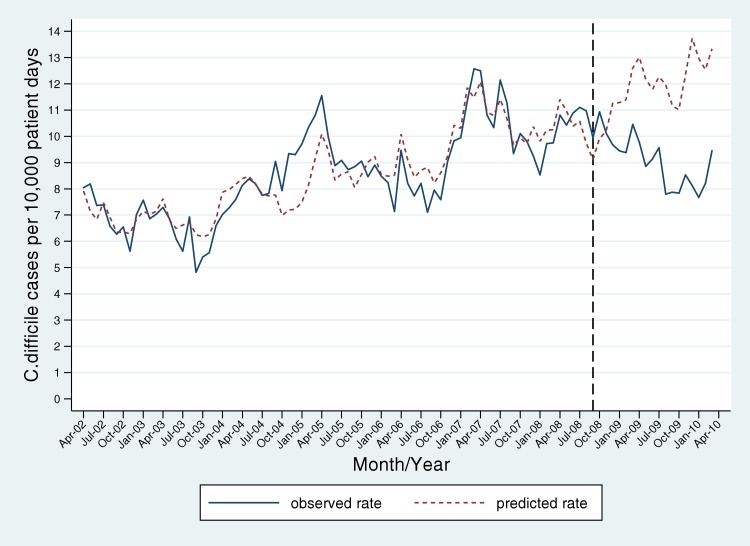
Reduced rates of *C. difficile* infection associated with the introduction of public reporting. Observed monthly rates of *C. difficile* infection in Ontario (solid blue line) were generally increasing prior to the introduction of public reporting in September 2008 (identified by black dotted line), and declined after this intervention. Post-intervention rates were significantly lower than rates predicted by a Poisson model (red dashed line) derived from pre-intervention data points and adjusted for age and hospital strata, and overall burden of community antibiotic use (with 0- to 12-mo lags).

After the introduction of public reporting, there was a gradual decline in age-specific hospital rates of *C. difficile* colitis in Ontario (Figure 3). The observed post-intervention rates diverged from predicted rates based on the pre-reporting-period trends: rates were forecasted to continue to rise during this period (Figure 3). There were 8,787 cases of *C. difficile* in Ontario during the 19-mo post-intervention period, as compared to 11,392 predicted by the Poisson model. In 2009, the first full calendar year after public reporting was instituted, there were only 5,417 cases (8.92 per 10,000 patient-days) as compared to 7,327 predicted by the Poisson model (12.16 per 10,000 patient-days, 95% CI 11.35–13.04 cases per 10,000 patient-days) (*p*<0.001). This corresponded to a 26.7% reduction in *C. difficile* cases (95% CI 21.4%–31.6%). Predicted rates were higher than observed rates across all facility types, including large community hospitals (12.9 versus 10.1 per 10,000 patient-days), small community hospitals (10.1 versus 5.4 per 10,000 patient-days), and acute teaching hospitals (11.1 versus 7.5 per 10,000 patient-days). Public reporting was associated with a projected 1,970 cases averted in the first calendar year after introduction (95% CI 1,476–2,500 cases) (Table 2). Findings were similar in a sensitivity analysis excluding two large hospitals with the greatest discordance between *C. difficile* rates in the publicly reported and administrative datasets (data not shown).

**Table 2:** Cases of *C. difficile* averted by public reporting based on differences in observed and expected monthly case counts.

Post-Intervention Month	Rate Ratio^a^ (95% CI)	Number of Person-Days	Number of Expected Cases of *C. difficile* b (95% CI)	Number of Observed Cases of *C. Difficile*	Number of Averted Cases of *C. Difficile* (95% CI)
Sep 2008	1.07 (0.95–1.21)	487,322	452 (401–510)	485	−33 (−84 to 25)
Oct 2008	1.10 (0.98–1.23)	526,840	523 (467–590)	576	−53 (−109 to 14)
Nov 2008	0.99 (0.86–1.13)	500,273	510 (447–585)	506	4 (−59 to 79)
Dec 2008	0.86 (0.76–0.96)	513,768	579 (517–654)	497	82 (20–157)
Jan 2009	0.83 (0.74–0.94)	534,666	603 (534–685)	505	98 (29–180)
Feb 2009	0.82 (0.73–0.92)	493,535	562 (504–634)	463	99 (41–171)
Mar 2009	0.83 (0.73–0.93)	534,744	674 (601–764)	559	115 (42–205)
Apr 2009	0.75 (0.66–0.84)	511,188	665 (592–753)	500	165 (92–253)
May 2009	0.73 (0.64–0.82)	505,739	615 (545–698)	448	167 (97–250)
Jun 2009	0.77 (0.69–0.87)	516,594	609 (545–683)	472	137 (73–211)
Jul 2009	0.78 (0.69–0.87)	508,844	624 (558–702)	487	137 (71–215)
Aug 2009	0.65 (0.57–0.74)	466,992	559 (492–643)	364	195 (128–279)
Sep 2009	0.70 (0.62–0.80)	480,041	536 (473–613)	378	158 (95–235)
Oct 2009	0.71 (0.62–0.81)	515,581	568 (497–652)	404	164 (93–248)
Nov 2009	0.69 (0.60–0.79)	503,973	622 (544–717)	430	192 (114–287)
Dec 2009	0.59 (0.51–0.68)	501,327	689 (601–797)	407	282 (194–390)
Jan 2010	0.59 (0.51–0.68)	516,159	669 (582–777)	396	273 (186–381)
Feb 2010	0.65 (0.57–0.75)	485,781	610 (535–701)	399	211 (136–302)
Mar 2010	0.71 (0.62–0.81)	540,613	721 (630–827)	511	210 (119–316)
Total post-intervention period			11,392	8,787	2,605

a Relative risks for post-intervention months are observed over expected *C. difficile* counts, where expected count is based on the Poisson model predictions for that month.

b Expected counts are based on Poisson model predictions for that month, adjusting for hospital, age strata, calendar month, and population antibiotic consumption with lags of 0–12 mo.

### Tracer Outcomes Not Expected to Be Impacted by *C. difficile* Public Reporting

**Figure 4:**
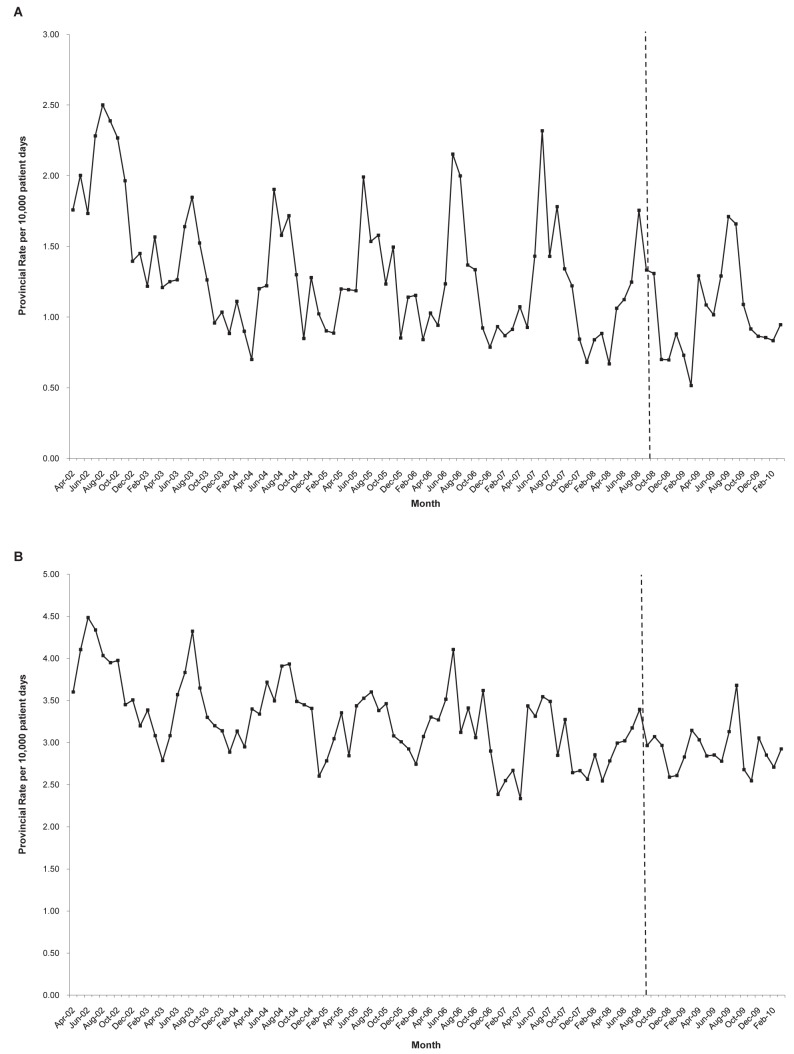
Tracer analyses evaluating longitudinal time trends of infections not expected to be impacted by hospital public reporting of *C. difficile* infection rates. Neither community-acquired bacterial gastrointestinal infections (A) nor urinary tract infections (B) exhibited a change in incidence concurrent with the introduction of *C. difficile* infection public reporting in September 2008.

During the study period (April 1, 2002 to March 31, 2010) there were 6,545 hospital admissions for other leading bacterial gastrointestinal pathogens, corresponding to a rate of 0.87 per 1,000 hospital admissions, or 1.34 per 10,000 patient-days. The rate of these predominantly community-acquired infections did not change after the introduction of *C. difficile* public reporting (Figure 4A).

There were 16,601 hospital admissions involving urinary tract infections, corresponding to a rate of 2.21 per 1,000 admissions, or 3.40 per 10,000 patient-days. Longitudinal trends in urinary tract infections did not change after the introduction of *C. difficile* public reporting (Figure 4B).

## Discussion

This longitudinal population-based cohort study has confirmed an immense burden of *C. difficile* infection in Ontario, while heralding mandatory hospital reporting as one potential means to reduce this burden. *C. difficile* infections affected more than 6,000 patients per year in Ontario, were more than twice as common as all other bacterial gastrointestinal and urinary tract infections combined, and increased over the 6.5-y pre-intervention period. However, with the introduction of public reporting in September 2008, *C. difficile* infections declined by 26% across Ontario, resulting in over 1,900 cases averted per year.

A number of jurisdictions in the United States, Canada, and the United Kingdom have introduced mandatory public reporting of hospital-acquired infections, including *C. difficile* colitis, and some have reported reductions in rates [24],[25]. However, our study represents the first attempt, to our knowledge, to measure population-based rates in a dataset that is independent from the public reporting system. In fact, a prior systematic review identified no rigorous studies investigating changes in health care–associated infection as an outcome of public reporting [12]. Therefore, our findings provide important confirmation that public reporting can be associated with reductions in health care–associated infections on a broad scale.

Although we did not explore the mechanisms by which public reporting influenced *C. difficile* rates in this case, prior research suggests that the selection pathway (i.e., patients selecting higher performing institutions) is least likely to have impacted *C. difficile* rates in Ontario. A recent review suggests that the impact of public reports on consumers is related to the accessibility and ease of understanding the messages of the report [26]. However, in Ontario the reports are quite deeply buried on a MOHLTC website [27]. So, even though the universal health care system in Ontario does not constrain individuals from choosing their own providers and institutions, it is unlikely that these reports led to shifts in patient selection of hospitals. It is more likely that public reporting elevated *C. difficile* to greater prominence on hospital quality improvement agendas, and motivated hospitals to adhere more closely to best practices in *C. difficile* prevention. Such practices, ranging from patient isolation to environmental cleaning, are well described in general infectious diseases society guidelines [6], and analogous Ontario guidelines were distributed to all hospitals. No financial incentives or disincentives were initially linked to *C. difficile* public reporting in Ontario, but hospitals may have anticipated that at some future point high rates could influence hospital reimbursement, akin to the US Centers for Medicare & Medicaid Services' denial of hospital reimbursement for avoidable patient complications such as infection (termed “never events”) [28]. In fact, subsequent legislation in Ontario has now mandated that executive compensation be linked to achieving quality improvement targets, including for *C. difficile* rates, starting in 2012 [14].

This investigation confirmed prior findings that higher *C. difficile* rates are strongly associated with older patient age [1],[29], large community hospitals [30], and winter (respiratory virus) season [30],[31]. Although prior antibiotic exposure is widely recognized as a cause of *C. difficile* colitis for individual patients (by disrupting normal intestinal flora) [1],[32], we report a novel finding that population-level outpatient antimicrobial consumption is predictive of hospital *C. difficile* rates across a broad geographic region. Overall antibiotic prescription rates predicted *C. difficile* rates at lags of up to 9 mo. Seasonal increases in antibiotic use in winter appear to drive seasonal increases in *C. difficile* disease, and likely mediate the reported association between influenza and *C. difficile* seasonality [31]. Intriguingly, the apparent increases in *C. difficile* rates in Ontario between 2002 and 2008 are also potentially explained by corresponding increases in population antibiotic utilization. This finding provides strong support for the “antibiotic stewardship” movement, aimed at reducing unnecessary antibiotic use within institutional and community settings [33].

Although study strengths include a population-based assessment of a system-level intervention with no loss to follow-up, as an observational study using health administrative data our study has some limitations. Our study findings may be influenced by misclassification of *C. difficile* outcome status in the administrative databases. However, the accuracy of these diagnostic codes are supported by patient-level validation studies in other jurisdictions [17],[18], as well as our own hospital-level cross-validation with Ontario public reporting statistics. Without a prospective, randomized trial we cannot be certain that *C. difficile* rates in Ontario were not influenced by some other temporal confounder, such as changes in *C. difficile* strain prevalence or antibiotic stewardship practices unrelated to *C. difficile* public reporting. However, the causal inference is strengthened by the duration of our longitudinal cohort (crude rates were rising for 6.5 y and then dropped coinciding with the intervention), the inclusion of crucial predictors of *C. difficile* (age and antibiotic utilization), and the assessment of control infectious diseases. Although public reporting could prompt some hospitals to underestimate the proportion of cases that were nosocomial, these hospitals are not likely to have underestimated the overall number of cases, since these figures were not subject to public reporting, and because ignoring cases or reducing laboratory testing of *C. difficile* could potentially lead to increased transmission of infection and more dramatic outbreaks. Nevertheless, given the potential for “gaming” of reported rates, and the potential for more thorough reporting in higher performing institutions, we utilized a data source for hospital *C. difficile* rates that was independent of the public reporting system itself.

This study provides to our knowledge the first population-based, rigorous evaluation of a public reporting system for hospital-acquired infection using an independent data source. In doing so, it provides support for ongoing public reporting of hospital *C. difficile* rates as a means of reducing the large population burden of this preventable disease. Future research will be required to discern the direct mechanism by which *C. difficile* infection rates are reduced in response to public reporting.

## Abbreviations

ICD: *International Classification of Diseases*
MOHLTC: Ontario Ministry of Health and Long-Term Care
